# A Large Video Set of Natural Human Actions for Visual and Cognitive Neuroscience Studies and Its Validation with fMRI

**DOI:** 10.3390/brainsci13010061

**Published:** 2022-12-29

**Authors:** Burcu A. Urgen, Hilal Nizamoğlu, Aslı Eroğlu, Guy A. Orban

**Affiliations:** 1 Department of Psychology, Bilkent University, 06800 Ankara, Türkiye; 2 Interdisciplinary Neuroscience Graduate Program, Bilkent University, 06800 Ankara, Türkiye; 3 Aysel Sabuncu Brain Research Center and National Magnetic Resonance Research Center (UMRAM), Bilkent University, 06800 Ankara, Türkiye; 4 Department of Psychology, Justus Liebig University in Giessen, 35394 Giessen, Germany; 5 Department of Medicine and Surgery, University of Parma, 43125 Parma, Italy

**Keywords:** vision, action observation, fMRI, action videos, naturalistic stimuli, cognitive neuroscience, systems neuroscience

## Abstract

The investigation of the perception of others’ actions and underlying neural mechanisms has been hampered by the lack of a comprehensive stimulus set covering the human behavioral repertoire. To fill this void, we present a video set showing 100 human actions recorded in natural settings, covering the human repertoire except for emotion-driven (e.g., sexual) actions and those involving implements (e.g., tools). We validated the set using fMRI and showed that observation of the 100 actions activated the well-established action observation network. We also quantified the videos’ low-level visual features (luminance, optic flow, and edges). Thus, this comprehensive video set is a valuable resource for perceptual and neuronal studies.

## 1. Introduction

One of the most important skills social organisms possess is the ability to perceive the actions of conspecifics in their environment. This skill has a survival value since it allows the organisms to take the appropriate action based on what they perceive. For instance, if somebody smiles at you, you probably smile back but if that person is about to attack you, you will probably want to flee. Therefore, the perceptual and neural mechanisms of action perception have received great interest from psychologists, systems, and cognitive neuroscientists.

Although there is a growing body of research in observed action processing [1], the range of actions used in such empirical studies has been limited. Since the beginning of 1990s, the majority of neuroscience studies have used *grasping* as the exemplary action to study action observation [1]. More recent work has extended this work by introducing other action categories, such as locomotion actions (e.g., walking), communicative actions (e.g., gestures), self-directed actions (e.g., scratching one’s own body), interaction actions (e.g., hugging), vocal actions (e.g., singing), or other manipulative actions (e.g., pushing an object) [2,3,4,5]. This body of work has demonstrated the significance of studying action categories other than grasping as the neural activations for different actions were localized in different brain regions especially at the level of parietal cortex. However, each individual study created its own limited set of action videos, and it has been difficult to make comparisons across studies due to variations in terms of actors, scenes, video durations, and video quality (e.g., frame per seconds or resolution). Given the richness of the human action repertoire, the aim of the current study is to introduce a large video set of actions performed in natural settings, covering the entire human repertoire and make it available to researchers working primarily in the fields of visual, cognitive neuroscience and psychology. We believe that such an effort is necessary and timely given recent work, indicated above, that shows differences in neural representations of different actions [2,3,4,5], the move to use more naturalistic stimuli in neuroscience studies [6,7], and the availability of multivariate pattern analysis techniques to analyze experimental data that has a rich set of conditions, i.e., a wide variety of actions [8], instead of a few of them, as is traditionally done in previous studies. It is important to note that in creating this database, we aim to address the shortcomings of earlier attempts, such as those that provided action stimuli in the form of point-light displays (See Table 1 in [9], as they do not have high ecological validity, and the ones that come from the computer vision community ([10,11]), as they are usually unconstrained in terms of actors, context, camera angle, and movements.

The action set presented in the current paper has two major features. It represents, as far as we know, the first attempt in visual and cognitive neuroscience to systematically encompasses the entire human behavioral repertoire, with the exception of emotion-driven actions (e.g., sexual) and those involving implements (e.g., using a tool or driving a car). Second, it aims to concentrate on actions that are evolutionarily old, i.e., actions to which the human brain would have adapted during evolution. Thus, all videos were recorded in natural settings (beaches, parks, riverbanks) avoiding artificial structures in the background, and using natural objects as targets of the actions (e.g., we used stones, fruits, and pieces of wood instead of man-made items). These specifications define a homogeneous group of action exemplars and distinguish our set from the large set of 80 atomic actions, collected by the Google research group from the internet [12].

Crucially, the action exemplars in this set are unrelated to any a-priori categories. Such a stimulus-driven approach, inspired by an fMRI study of voxel-level selectivity for the meanings of words [13], can be considered complementary to the earlier studies in which the action exemplars were selected a priori as part of a single class (e.g., dragging, grasping, dropping, and pushing considered to be manipulative actions in earlier studies) [3,5,14]. On the other hand, it is important to note that, unlike the studies that use continuous natural movies (e.g., [13,15]), our stimuli set includes human actions without the clutter of other movements such as those of objects or other actors. Therefore, it constitutes a more suitable dataset for researchers who would like to study visual action perception and processing in humans.

## 2. Materials and Methods

In this section, we describe the stimulus set and the post-processing of the videos. The stimulus set is freely available and can be downloaded from https://osf.io/u62bp/?view_only=393a2924aa05461394fe9f3171863b94 (accessed on 29 December 2022). We also carried out a validation study with fMRI to show that our stimuli drive the regions established to be associated with the processing of actions, also known as the Action Observation Network [1,7,8]. The fMRI data can also be downloaded from the same link above.

### 2.1. Stimulus Set

***Actors***: Four actors performed the actions (2 males, 2 females). One additional female actor accompanied some actors in videos portraying actions that involved two individuals. They were undergraduate students at the University of Parma. We did not choose professional actors for two reasons. First, we wanted to record actions that were as natural as possible, without stylized movements that could be introduced by professional actors. Second, we wanted to have variability in the body movements which reflected individual differences. Before the recording of each action video, all actors were directed concerning how to perform the action and instructed to perform the action as naturally as possible within 3 s. For each action, several recordings were taken one after the other to make sure that the action was performed as intended in terms of timing and naturalness, and the best recording was chosen during the post-processing of videos. The actors were paid for their participation in the recordings and gave informed consent for their videos to be published in scientific journals.

**Figure 1:**
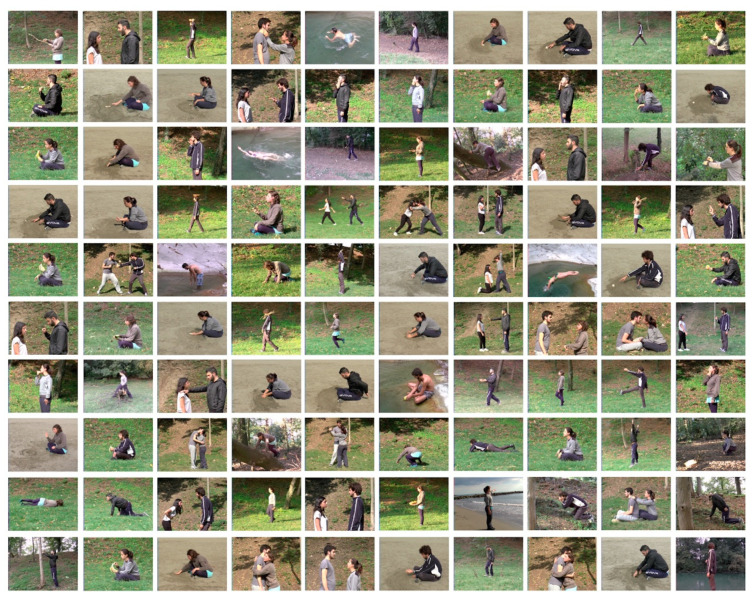
Sample frames from the stimulus set, one frame from each action exemplar. The first row corresponds to action exemplars 1–10, the second row corresponds to action exemplars 11–20, and so on. The action numbers refer to the numbers in Table 1.

***Actions***: One hundred different actions were recorded for several seconds (at least 3 s) and each action was performed by 3 of the 4 actors. So, in total, we recorded 300 videos with a fixed camera. Actions involved various effectors including the fingers, hand, arm, foot, mouth, upper body, or full body. One or 2 actors were portrayed in each video. When two actors were present, one could target the other with his action, or the two could interact. The actions were recorded from a lateral viewpoint. A sample frame from each video is presented in Figure 1. Table 1 lists the actions shown in the 100 videos and the actors who perform each of them. The different actions are described in the Supplementary Information.

### 2.2. Post-Processing of the Videos

The videos were recorded using Panasonic HCX 900 camcorders. After recording, the videos were edited using Final Cut Pro software and 3-s clips were made. The frame rate of these videos was 50 fps, so each video consisted of 150 frames. The size (height and width) of the frames was set to 314 × 410 pixels. The 3-s videos were then exported in .avi format and compressed using MPlayer’s *mencoder* command (http://www.mplayerhq.hu/, accessed on 29 December 2022).

The videos portray different action exemplars in natural settings, which entails variations in low-level features such as luminance, motion, or edges. We quantified those variables for each video. Hence, they can be used as variables of no interest in the experimental designs to minimize the effects of such low-level factors.

### 2.3. Data Validation

We validated our stimuli set with an fMRI experiment. Four human subjects participated in our study (2 females and 2 males; Mean Age: 26.5). Ethical approval was received from the Human Research Ethics Committee of Bilkent University.

#### 2.3.1. fMRI Experiment

Each participant underwent two fMRI sessions, each having 8 runs. In each session, the 100 action exemplar videos were split across odd and even runs. In each of the odd runs (i.e., runs one, three, five, and seven), the first 50 action exemplars were shown in a random order as mini-blocks of three video versions of the same action presented consecutively. Each video lasted 3 s, and thus, each mini-block was shown for a total of 9 s. In each of the even runs (i.e., runs two, four, six, and eight), the other 50 action exemplars were shown as randomly ordered mini-blocks of three video versions as in the odd runs. So, in total, each of the 100 action exemplars was presented 24 times across the two sessions. The order of the mini-blocks and video versions in each mini-block was randomized across different runs. An inter-stimulus interval ranging between 1–2 s was included in between the mini-blocks. The total duration of each run was 553.36 s.

In order to keep their attention throughout the runs, a question was asked in each repetition cycle about the video that was just presented (e.g., “Was it climbing up a tree?”) with a simple yes or no button-press response time period of 3 s. The periods of question were not included in the analysis.

#### 2.3.2. fMRI Data Acquisition

Participants were scanned at National Magnetic Resonance Research Center (UMRAM) in Bilkent University by using a 3T Siemens TimTrio MR scanner with a 32-channel phase array head coil. In order to minimize head movement, relevant foam paddings were put under their skull, around their neck, and under their legs. Stimuli were presented on an MR-compatible LCD screen (TELEMED, 60 Hz refresh rate, 800 × 600 pixel, 32 inches) and seen through a mirror system mounted on top of the head coil that is 168 cm away.

A high-resolution T1-weighted anatomical image covering the entire brain was acquired before the functional scans using the following acquisition parameters: TE = 2.92 ms, TR = 2.6 s, flip angle = 12°, Acceleration factor = 2, 176 sagittal slices with 1 mm × 1 mm × 1 mm resolution). Later on, for each of the eight experimental runs, functional images were acquired using echo-planar imaging (EPI) sequence (TR = 3 s, TE = 30 ms, flip angle = 90°, 96 × 96 matrix with FOV 240, 49 horizontal slices with 2.5 mm slice thickness). Each run started with the collection of 5 dummy scans to ensure that MR signal reached a steady state.

#### 2.3.3. fMRI Data Preprocessing

Results included in this paper are based on fMRI data preprocessed using fMRIPrep 20.1.1 ([9]; RRID:SCR_016216), which is based on Nipype 1.5.0 ([10]; RRID:SCR_002502).

##### Anatomical Data Preprocessing

A total of 2 T1-weighted (T1w) images were found within the input BIDS dataset. All of them were corrected for intensity non-uniformity (INU) with N4BiasFieldCorrection [16], distributed with ANTs 2.2.0 ([17], RRID:SCR_004757). The T1w-reference was then skull-stripped with a Nipype implementation of the antsBrainExtraction.sh workflow (from ANTs), using OASIS30ANTs as target template. Brain tissue segmentation of cerebrospinal fluid (CSF), white-matter (WM), and gray-matter (GM) was performed on the brain-extracted T1w using fast (FSL 5.0.9, RRID:SCR_002823, [18]). A T1w-reference map was computed after registration of 2 T1w images (after INU-correction) using mri_robust_template (FreeSurfer 6.0.1, [19]). Brain surfaces were reconstructed using recon-all (FreeSurfer 6.0.1, RRID:SCR_001847, [20]), and the brain mask estimated previously was refined with a custom variation of the method to reconcile ANTs-derived and FreeSurfer-derived segmentations of the cortical gray-matter of Mindboggle (RRID:SCR_002438, [21]). Volume-based spatial normalization to one standard space (MNI152NLin2009cAsym) was performed through nonlinear registration with antsRegistration (ANTs 2.2.0), using brain-extracted versions of both T1w reference and the T1w template. The following template was selected for spatial normalization: ICBM 152 Nonlinear Asymmetrical template version 2009c ([22], RRID:SCR_008796; TemplateFlow ID: MNI152NLin2009cAsym).

##### Functional Data Preprocessing

For each of the 16 BOLD runs recorded per subject (across all sessions), the following preprocessing was performed. First, a reference volume and its skull-stripped version were generated using a custom methodology of *fMRIPrep*. The BOLD reference was then co-registered to the T1w reference using bbregister (FreeSurfer) which implements boundary-based registration [23]. Co-registration was configured with six degrees of freedom. Head-motion parameters with respect to the BOLD reference (transformation matrices, and six corresponding rotation and translation parameters) are estimated before any spatiotemporal filtering using mcflirt (FSL 5.0.9, [24]). BOLD runs were slice-time corrected using 3dTshift from AFNI 20160207 ([25], RRID:SCR_005927). The BOLD time-series were resampled to surfaces on the following spaces: *fsaverage5*. The BOLD time-series (including slice-timing correction when applied) were resampled onto their original, native space by applying a single, composite transform to correct for head-motion and susceptibility distortions. These resampled BOLD time-series will be referred to as *preprocessed BOLD in original space*, or just *preprocessed BOLD*. The BOLD time-series were resampled into standard space, generating a *preprocessed BOLD run in [‘MNI152NLin2009cAsym’] space*. First, a reference volume and its skull-stripped version were generated using a custom methodology of *fMRIPrep*. Several confounding time-series were calculated based on the *preprocessed BOLD*: framewise displacement (FD), DVARS and three region-wise global signals. FD and DVARS are calculated for each functional run, both using their implementations in *Nipype* (following the definitions by [26]). The three global signals are extracted within the CSF, the WM, and the whole-brain masks. Additionally, a set of physiological regressors were extracted to allow for component-based noise correction (*CompCor*, [27]). Principal components are estimated after high-pass filtering the preprocessed BOLD time-series (using a discrete cosine filter with 128s cut-off) for the two *CompCor* variants: temporal (tCompCor) and anatomical (aCompCor). tCompCor components are then calculated from the top 5% variable voxels within a mask covering the subcortical regions. This subcortical mask is obtained by heavily eroding the brain mask, which ensures it does not include cortical GM regions. For aCompCor, components are calculated within the intersection of the aforementioned mask and the union of CSF and WM masks calculated in T1w space, after their projection to the native space of each functional run (using the inverse BOLD-to-T1w transformation). Components are also calculated separately within the WM and CSF masks. For each CompCor decomposition, the *k* components with the largest singular values are retained, such that the retained components’ time series are sufficient to explain 50 percent of variance across the nuisance mask (CSF, WM, combined, or temporal). The remaining components are dropped from consideration. The head-motion estimates calculated in the correction step were also placed within the corresponding confounds file. The confound time series derived from head motion estimates and global signals were expanded with the inclusion of temporal derivatives and quadratic terms for each [28]. Frames that exceeded a threshold of 0.5 mm FD or 1.5 standardized DVARS were annotated as motion outliers. All resamplings can be performed with a *single interpolation step* by composing all the pertinent transformations (i.e., head-motion transform matrices, susceptibility distortion correction when available, and co-registrations to anatomical and output spaces). Gridded (volumetric) resamplings were performed using antsApplyTransforms (ANTs), configured with Lanczos interpolation to minimize the smoothing effects of other kernels [29]. Non-gridded (surface) resamplings were performed using mri_vol2surf (FreeSurfer).

Many internal operations of fMRIPrep use Nilearn 0.5.2 ([30], RRID:SCR_001362), mostly within the functional processing workflow. For more details of the pipeline, see the section corresponding to workflows in fMRIPrep’s documentation.

#### 2.3.4. Activation Maps for Observed Actions

An important aim of the present study is to show that our novel stimuli set could drive the action observation network as previously identified, and to investigate whether it could be extended given that previous work used a limited number of actions. This is achieved by univariate analysis, which reveals the activation map of actions.

Following pre-processing, we ran a general linear model (GLM) composed of 8 regressors, including 1 regressor for all the action videos, 6 motion regressors (3 translations and 3 rotations), and a constant factor. All regressors were convolved with the default canonical hemodynamic response function in SPM12. The activation map was generated by the beta value corresponding to the action videos.

#### 2.3.5. Definition of ROIs

**Figure 2:**
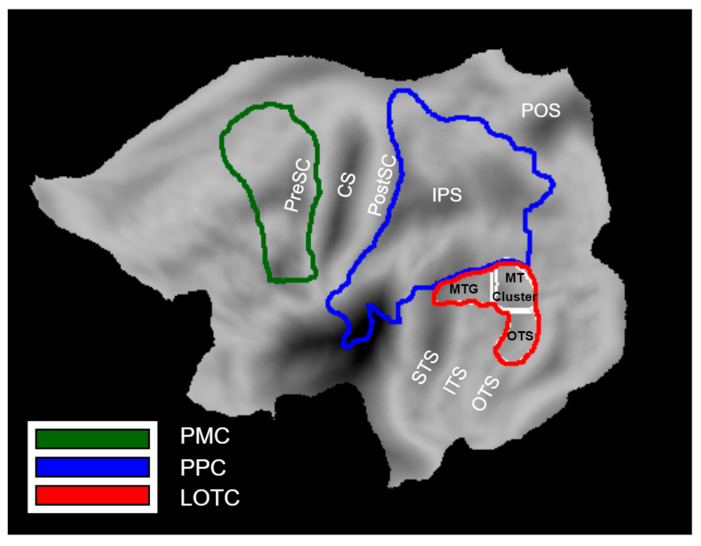
A priori ROIs of the Action Observation Network: LOTC (yellow line), PPC (blue line), PMC (green line). LOTC consists of three sub-regions including the MT cluster, MTG, and OTS. The anatomical landmarks are indicated in black: CS (Central sulcus), PreSC (Pre-central sulcus), PostCS (Post-central sulcus), IPS (intra-parietal sulcus), POS (parieto-occipital sulcus), STS (superior temporal sulcus), ITS (inferior temporal sulcus), OTS (occipito-temporal sulcus).

We defined three a priori ROIs that represent the three levels of the Action Observation Network based on previous work: Lateral occipito-temporal cortex (LOTC), posterior parietal cortex (PPC), and premotor cortex (PMC) (Figure 2). LOTC included the regions of the action observation network based on the activation map (threshold at *p* < 0.001) of [3]. These regions included (1) the MT cluster [31,32], (2) a portion extending dorsally from the MT cluster onto the middle temporal gyrus referred to as MTG, and (3) a portion extending ventrally from the MT cluster onto occipital temporal sulcus that we refer to as OTS. The MTG and OTS correspond to the upper bank and lower bank of the macaque STS anterior to the MT cluster [33], the two regions which project to the parietal level of the action observation network in the macaque [34]. They are therefore considered to be input regions for the next level, PPC.

The PPC included the cyto-architectonic regions of SPL, IPS, IPL, and parietal operculum [35,36,37,38]. Its posterior boundary coursed between V6 and V6A [39] and extended between V3D and V7 [40].

The PMC included the cyto-architectonic supplementary, dorsal, and ventral premotor areas taken from the Anatomy software, but the ventral part was extended in the rostral direction to include regions that are responsive to observed actions according to [41,42].

All ROIs were defined on flat maps in Caret software [43]. Only ROIs in the left hemisphere were considered, because we now have considerable evidence that the position of the actor in the visual field affects the lateralization of PPC activation in the action observation paradigm [41], with the activation being contralateral to the hemifield in which the actor is shown. In almost all videos (with only a few exceptions represented by the 2 swimming exemplars, diving, and rolling side-way), the body of the actor was either motionless in the right visual field or remained in this field where actions (e.g., walking) implied horizontal motion to the left, as the camera partially followed the action.

## 3. Results

### 3.1. Post-Processing of Video Stimuli

We quantified low-level visual features for each video, including luminance, motion, and edges. These features can be used as variables of no interest in fMRI experiments to minimize the effects of those low-level factors. The MATLAB codes that generate these features as well as the output of these codes for each video can be downloaded from https://osf.io/u62bp/?view_only=393a2924aa05461394fe9f3171863b94 (accessed on 29 December 2022).

**Figure 3:**
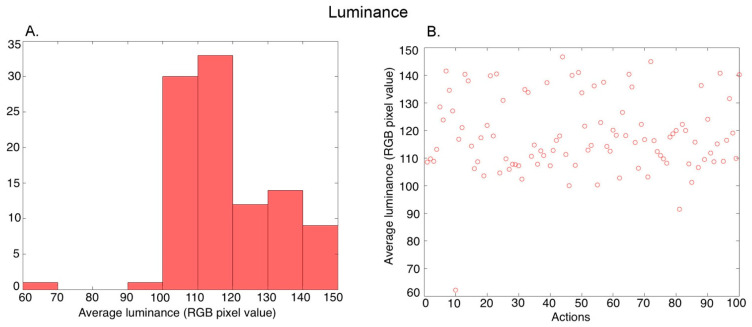
Characterization of the average luminance in the videos. (**A**) Histogram of the average luminance values across 100 action exemplars (averaged over the 3 versions of each action performed by different actors), (**B**) Average luminance of the 100 action exemplars.

***Luminance***: For a given video, we first found the average of the RGB pixel values in all 150 frames (temporal averaging), and then calculated the average of the pixels in the averaged frame (spatial averaging). Thus, we obtained a single luminance value characterizing each video. Figure 3A shows the histogram of the average luminance values over the 100 action exemplars (further averaged over the three versions of an exemplar performed by different actors), and Figure 3B shows the values for the different actions.

**Figure 4:**
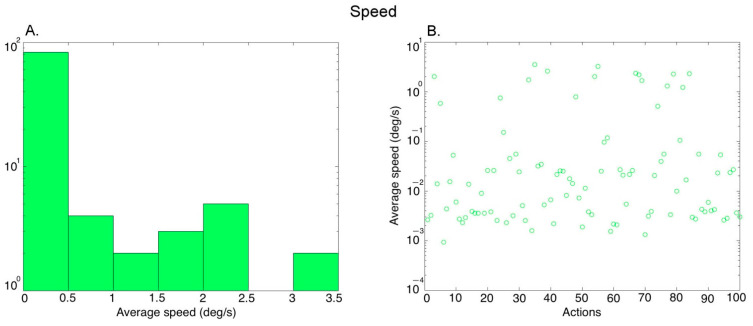
Characterization of the average speed in the videos. (**A**) Histogram of the average speed values across 100 action exemplars (averaged over the 3 versions of each action performed by different actors), (**B**) Average speed of the 100 action exemplars.

***Motion***: We computed the mean speed in each video using an algorithm by [44]. The local motion vector was computed for each pixel in the image on a frame-by-frame basis. We performed temporal averaging (across frames) and spatial averaging (within a frame) (see Luminance above) to obtain one value for each video. Figure 4A shows the histogram of the average speed values covering the 100 action exemplars (further averaged over the three versions of each exemplar performed by different actors), and Figure 4B shows the values for the different actions.

**Figure 5:**
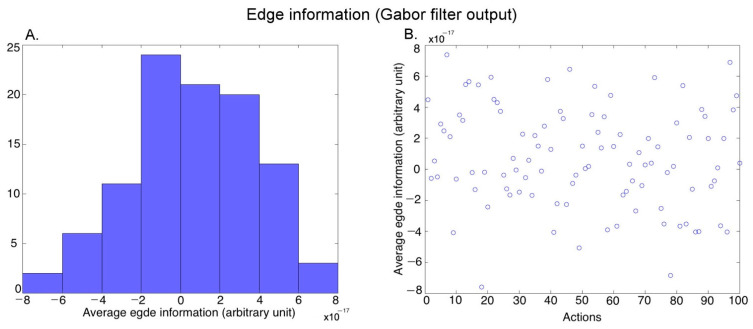
Characterization of the average edge information in the videos. (**A**) Histogram of the average edge information across 100 action exemplars (averaged over the 3 versions of each action performed by different actors), (**B**) Average edge information of the 100 action exemplars.

***Edges (Form)***: We passed each video frame of each action exemplar through a set of Gabor filters [45] to extract the edge information. We used Gabors of 5 scales and 8 orientations using the Gabor filtering algorithm described in [46]. We performed temporal averaging (across frames) and spatial averaging (within a frame) (see Luminance above) to obtain one output value for each video. Figure 5A shows the histogram of the average edge information covering the 100 action exemplars (further averaged over the three versions of an exemplar performed by different actors), and Figure 5B shows the edge information for the different actions.

### 3.2. fMRI Activation Maps

**Figure 6:**
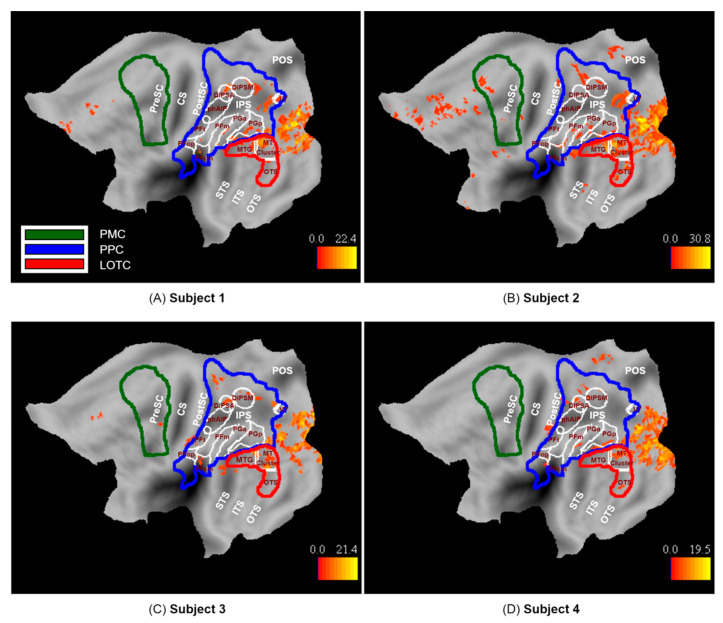
Activation map for observed actions (*p* < 0.001 uncorrected, k = 10) on the flattened left hemisphere of subjects (**A**) 1, (**B**) 2, (**C**) 3, and (**D**) 4.

Observation of the 100 actions used in the present study activated the three levels of the action observation network (*p* < 0.001 uncorrected): LOTC, PPC, and to a lesser degree PMC in the left hemisphere, as expected (Figure 6). The LOTC level included activations in the MT cluster as well as MTG and to some extent OTS, as previously defined (See Section 2.3.5). The PPC level included activations in functionally defined areas DIPSM in all participants and DIPSA in subjects 1, 2, and 4. Other PPC level activations in cyto-architectonic areas of the inferior parietal lobule (IPL) include PFcm, PGa, and PGp in subject 1; PFcm, PFm, PGa, and PGp in subject2; PFcm and PFt in subject 3, and finally PFop, PGa, and PGp in subject 4. In addition, superior parietal lobule areas in dorsal postcentral gyrus were activated in subjects 2, 3, and 4. The PMC level included areas in the anterior part of the dorsal premotor and ventral premotor cortex in Subject 2 and the posterior part of the ventral premotor cortex in Subject 3.

In addition to the ROIs of the action observation network, several other areas were activated by the observation of actions. These included mainly the early visual cortex, extending into the neighboring parieto-occipital sulcus, including V7 [47], in Subject 1. Additional activations included a medial frontal site in Subjects 1, 2, and 3 and another small cluster neighboring the parieto-occipital sulcus (POS) in Subjects 2 and 4.

## 4. Discussion

We describe a video stimulus set that consists of 100 different natural actions covering most of the human repertoire. Each action is performed by multiple actors. The low-level features of the videos were quantified, allowing them to be factored out in future experiments. Our fMRI validation study showed that the novel stimuli presented here drove the action observation network. The weak activation of the premotor level likely reflects a combination of two factors: a lower level of activation due to distance from the retina [34], as documented in many studies (e.g., [2]), and an increased selectivity of premotor voxels whereby these voxels are activated by only a small number of observed actions, typical for the present study.

To the best of our knowledge, this is the largest action database to be made available for use in psychology and cognitive neuroscience research. Earlier work used *grasping* as the exemplary action for a long-time in action observation research [1]. More recent work has introduced different action categories such as locomotion, communicative, self-directed, interaction, and vocal actions [2,3,4,5]. However, each study was constrained by a small set of action videos that was created for the purposes of that study and this made it difficult to compare the results across different studies due to variations in actors, scenes, video durations, and video quality. There are video databases that display actions in the form of point-light displays (See the list in [9]) to overcome the visual differences in the stimuli, but the shortcoming of these databases is that the stimuli are not naturalistic enough and lack ecological validity. There are some action databases with naturalistic actions, such as [48], but it focuses on actions that have emotional content. There are yet other naturalistic action databases, such as UCF50 [10] or HMDB51 [11], but their target is usually the computer vision community. Computer vision research has different constraints such as multiple cameras, camera motion, or clutter in the videos. Therefore, the databases have been created in accordance with the problems needing to be solved from a computer-vision perspective. Given that we are at the early stages of understanding the perceptual and neural mechanisms processing observed actions, it is necessary to initiate such studies using a stimulus set that is natural yet simple and sufficiently controlled to facilitate interpretation. In this respect, they differ from the set of 80 atomic actions [12].

We believe that our database will prove useful for researchers who intend to study the perceptual and neural differences between observing action exemplars such as locomotion or communicative actions, as well as interactions between two individuals, extending ongoing fMRI work in passive subjects with different action classes [2,3,4,5]. Our stimulus set can, however, be used in a much wider set of behavioral and neuroimaging studies, allowing some generic plausible models to be built for action perception. Indeed, our stimulus set can be used in an array of visual tasks. A first set are discrimination tasks, probing the identity of the observed actions, such as identification or same–different tasks [49,50]. If, as has been proposed [51], observed actions of different classes, such as manipulation or locomotion, are processed in different PPC regions, one would expect action discriminability, whether measured perceptually or in neural activity, to depend on the classes involved. A second set of tasks are classification studies probing the semantic categories of observed actions. The classification of static images has received a lot of attention comparing human and deep network performance, in an effort to model object processing in the ventral pathway [52]. This can be extended to the classification of videos to model observed action processing in the dorsal pathway. Yet another set of studies are similarity studies. Subjects have to rate how close two actions are, allowing to derive the distances between observed actions in perceptual space, which can be compared using RSA to the distance between these actions in a neural space derived from single cell recordings or fMRI activations. Such perceptual and neuronal studies would stimulate the computational modelling of observed actions. There have been a few modelling attempts to explain the neural mechanisms of observed actions [53,54,55,56], but so far these have been limited to only a few action exemplars such as locomotion or grasping and such modelling efforts would benefit from testing a larger set of actions. It is noteworthy that in the present stimulus set the actions were performed by several actors, which makes it easy to design control tasks, requiring subjects to discriminate or classify actors. Extending this further, the video set can also be used in studies for person or gender identification from body movements as we have multiple actors performing the same action with the same background.

A limitation of our stimuli set is that all actions take place in an outdoor scene. Therefore, researchers who are interested in contextual effects, such as the scene in which the action takes place, may not find sufficient variability in the stimulus set, although some actions were set on a beach, or in a lake, in addition to the grassy landscape. However, many of these actions can be performed indoors as well, and hence from an action-identity point of view, many of them can still be used in behavioral and neuroimaging experiments probing action observation.

Another limitation of the stimuli set is that we do not systematically control the emotional content of the actions. Most actions can be considered as neutral, such as locomotion, but some of them have an inherently positive valence, such as laughing, or negative valence, such as signing “no”. Therefore, the set may not be optimal for studies aiming to systematically investigate the emotional content of the actions.

## 5. Conclusions

In summary, we believe that our stimuli set will be beneficial to the scientific community studying action perception from behavioral, neuro-scientific, and computational perspectives, particularly those who wish to move away from mere grasping and reaching as prototypical actions. As the stimulus set can be combined not only with fMRI, but also MEG, EEG, and stereo-EEG, it should find a wide range of applications from the bedside to the laboratory.

## Figures and Tables

**Table 1:** Number and name of action exemplars, with the actors performing them (M1: male actor 1, M2: male actor 2, F1: female actor 1, F2: female actor 2).

Action Exemplar No	Action Exemplar Name	Actors
1	Measuring with fingers	F1, F2, M1
2	Shouting	F1, M1, M2
3	Carrying with head and hands	F1, M1, M2
4	Caressing another person	F1, F2, M1
5	Free style swimming	F2, M1, M2
6	Kicking wood with feet	F2, M1, M2
7	Dragging	F1, F2, M2
8	Reaching	F1, F2, M2
9	Measuring a long distance with feet	F1, F2, M1
10	Crushing a leaf with fingers	F1, F2, M2
11	Fanning with leaf	F1, F2, M2
12	Pushing a small stone	F1, F2, M2
13	Dropping a small stone	F1, F2, M2
14	Ridiculing another person	F1, F2, M1
15	Massaging own cheek	F1, F2, M2
16	Scratching own cheek	F1, F2, M2
17	Swallowing	F1, F2, M1
18	Yawning with hand	F1, F2, M2
19	Licking an orange	F1, F2, M2
20	Gazing at an object	F1, F2, M1
21	Peeling a fruit	F1, F2, M1
22	Filling a hole with hand	F1, F2, M1
23	Hitting own cheek	F1, F2, M1
24	Swimming back style	F1, M1, M2
25	Displacing wood	F1, F2, M2
26	Weighing an object with one hand	F1, F2, M2
27	Climbing down a tree	F1, F2, M1
28	Whistling	F2, M1, M2
29	Measuring with hands	F1, F2, M1
30	Picking a fruit from a tree	F2, M1, M2
31	Kicking horizontally	F2, M1, M2
32	Kicking vertically	F2, M1, M2
33	Carrying with head	F1, M1, M2
34	Blowing a leaf	F1, M1, M2
35	Chasing another person	F1, M1, M2
36	Struggling	F2, M1, M2
37	Waving goodbye	F1, M1, M2
38	Beating with a piece of wood	F1, M1, M2
39	Carrying with shoulder and hand	F1, F2, M2
40	Reprimanding a person	F2, M1, M2
41	Biting a banana	F2, M1, M2
42	Fighting with another person	F2, M1, M2
43	Washing own body	F2, M1, M2
44	Foraging	F1, M1, M2
45	Stretching own body	F1, M1, M2
46	Writing with fingers	F1, M1, M2
47	Charging to attack	F1, F2, M1
48	Diving	F2, M1, M2
49	Pointing nearby	F2, M1, M2
50	Squeezing an orange	F1, F2, M2
51	Forbidding with fingers	F2, M1, M2
52	Wrapping a stone	F1, M1, M2
53	Grasping	F2, M1, M2
54	Carrying on shoulder	F1, F2, M1
55	Running	F1, F2, M2
56	Burying in the sand	F2, M1, M2
57	Stopping a person	F1, F2, M2
58	Pushing a person	F1, M1, M2
59	Kissing a person	F1, F2, M1
60	Throwing and catching a small piece of wood	F1, M1, M2
61	Caressing own cheek	F2, M1, M2
62	Overtaking an obstacle	F1, F2, M1
63	Touching another person on the shoulder	F1, F2, M2
64	Building pyramid from sand	F1, F2, M1
65	Hiding an object behind back	F2, M1, M2
66	Washing fruit	F1, F2, M1
67	Carrying with hands	F1, F2, M2
68	Walking	F1, F2, M2
69	Marching	F1, F2, M1
70	Rubbing own cheek	F1, M1, M2
71	Throwing nearby	F1, M1, M2
72	Masticating	F2, M1, M2
73	People meeting	F2, M1, M2
74	Climbing up a tree	F1, F2, M2
75	Dancing with another person	F2, M1, M2
76	Getting up	F1, M1, M2
77	Crawling	F1, M1, M2
78	Spitting a piece of banana	F1, F2, M1
79	Doing gymnastics with both feet and arms	F1, F2, M2
80	Pushing a large object	F2, M1, M2
81	Rolling body sidewise	F1, F2, M1
82	Walking on hand and knees	F1, M1, M2
83	Laughing together with another person	F2, M1, M2
84	Carrying with one hand	F1, F2, M1
85	Singing a song	F1, F2, M1
86	Weighing an object with two hands	F1, M1, M2
87	Pointing distantly	F1, F2, M2
88	Drinking with hands	F2, M1, M2
89	Massaging another person	F1, F2, M1
90	Drinking with mouth	F1, F2, M2
91	Measuring height with own body	F1, M1, M2
92	Pinching off piece of banana	F1, F2, M1
93	Erasing	F1, F2, M2
94	Hugging a person (passive)	F1, M1, M2
95	Speaking with another person	F1, F2, M1
96	Rotating a stone	F2, M1, M2
97	Measuring a short distance with feet	F1, M1, M2
98	Hugging each other	F1, M1, M2
99	Digging a hole with a hand	F2, M1, M2
100	Throwing far	F1, F2, M1

## Data Availability

The data and stimuli presented in this study are available on Open Science Framework platform: https://osf.io/u62bp/?view_only=393a2924aa05461394fe9f3171863b94 (accessed on 29 December 2022).

## Supplementary Materials

The following supporting information can be downloaded at: https://www.mdpi.com/article/10.3390/brainsci13010061/s1.
