# Supplementary material for: A Large Video Set of Natural Human Actions for Visual and Cognitive Neuroscience Studies and Its Validation with fMRI
Source: Brain Sci. 2022 Dec 29;13(1):61. doi: 10.3390/brainsci13010061 (PMC9856703; doi:10.3390/brainsci13010061)
Supplement: Supplementary file 1 [file brainsci-13-00061-s001.zip › brainsci-2100387-supplementary.pdf]

SUPPLEMENTARY INFORMATION

A Large Video Set of Natural Human Actions for Visual and Cognitive Neuroscience Studies and Its Validation with fMRI

Burcu A. Urgen [1,2,3], Hilal Nizamoğlu [2,3,4], Aslı Eroğlu [2,3] and Guy A. Orban [5,*]

[1] Department of Psychology, Bilkent University, 06800 Ankara, Türkiye
[2] Interdisciplinary Neuroscience Graduate Program, Bilkent University, 06800 Ankara, Türkiye
[3] Aysel Sabuncu Brain Research Center and National Magnetic Resonance Research Center (UMRAM), Bilkent University, 06800 Ankara, Türkiye
[4] Department of Psychology, Justus Liebig University in Giessen, 35394 Giessen, Germany
[5] Department of Medicine and Surgery, University of Parma, 43125 Parma, Italy
* Correspondence: guy.orban@kuleuven.be

Action descriptions:

1. *Measuring with fingers*: The actor measures the length of a piece of wood using his/her fingers. He/she uses the right hand to hold the wood and the left hand to do the measurement. He/she stands on foot. The background is a park-like grassy area.
2. *Shouting:* Two individuals (one male, the other female) are facing each other. The one on the right shouts to the other one. The background is a grassy area.
3. *Carrying with head and hands*: The actor carries a basket on his head using both hands. He/she walks from right to left while carrying the basket. The background is a grassy area.
4. *Caressing another person*: Two individuals (one male, the other female) face each other. The one on the right brushes the right cheek of the other one rhythmically with his/her left hand. The background is a grassy area.
5. *Free style swimming*: The actor swims free style in a lake from right to left using both arms and legs.
6. *Kicking wood with feet*: The actor kicks a piece of wood with his/her right foot. The background is a grassy area.
7. *Dragging*: The actor sits on a sandy area (beach) and drags an object towards himself/herself using his/her right hand.
8. *Reaching*: The actor sits on a sandy area (beach) and reaches for an object with the right hand.
9. *Measuring a long distance with feet*: The actor measures the distance between two pieces of wood using his/her feet. He/she takes large steps from right to left to do the measurement. The background is a grassy area.
10. *Crushing a leaf with fingers*: The actor sits in a grassy area and crushes a leaf with his/her fingers (both hands).
11. *Fanning with leaf*: The actor sits in a grassy area and moves a leaf back and forth with the right hand to agitate the air.
12. *Pushing a small stone*: The actor sits on a sandy area (beach) and pushes a stone away with the right hand.

13. *Dropping a small stone*: The actor sits on a sandy area (beach) and drops a stone from the right hand to the ground.
14. *Ridiculing another person*: Two individuals (one male, the other female) face each other. The one on the right laughs at the one on the left, pointing with the right index finger. The background is a grassy area.
15. *Massaging own cheek*: The actor is standing in a grassy area and massages his/her left cheek using the right hand.
16. *Scratching own cheek*: The actor is standing in a grassy area and scratches his/her left cheek using the right hand.
17. *Swallowing*: The actor sits in a grassy area and holds a peeled banana in his/her left hand. He/she swallows the banana slowly.
18. *Yawning with hand*: The actor is standing in a grassy area, and yawns using his/her right hand.
19. *Licking an orange*: The actor sits in a grassy area, holds a half orange in his/her left hand, and licks it.
20. *Gazing at an object*: The actor sits on a sandy area (beach) and gazes at a stone on the sand.
21. *Peeling a fruit*: The actor sits in a grassy area, holding a banana in the left hand, and peels it with the right hand.
22. *Filling a hole with hand*: The actor sits on a sandy area (beach) and fills a hole with his/her hands.
23. *Hitting own cheek*: The actor is standing in a grassy area and hits his/her left cheek with the right hand rhythmically.
24. *Swimming backstroke*: The actor swims backstroke in a lake from right to left.
25. *Displacing wood*: The actor takes a piece of wood from the ground and moves it closer with the left hand.
26. *Weighing an object with one hand*: The actor is standing in a grassy area and weighs a piece of wood with his/her left hand.
27. *Climbing down a tree*: The actor climbs down a tree using his/her hands and feet.
28. *Whistling*: Two individuals (one male, the other female) face each other. The one on the right whistles towards the one on the left. The background is a grassy area.
29. *Measuring with hands*: The actor measures a large piece of wood with his/her hands.
30. *Picking a fruit from a tree*: The actor holds a branch of a tree with his/her left hand and picks a fruit from it with the right hand.
31. *Kicking horizontally*: The actor sits on a sandy area (beach) and kicks a small object with his/her hand horizontally over the ground.
32. *Kicking vertically*: The actor sits on a sandy area (beach) and knocks a small object with his/her hand vertically up.
33. *Carrying with head*: The actor carries a basket on his head without using the hands. He/she walks from right to left while carrying the basket. The background is a grassy area (like a national park).
34. *Blowing a leaf*: The actor sits in a grassy area, holds a leaf in his/her left hand, and blows towards the leaf.
35. *Chasing another person*: Two actors participate in the scene: one male, one female. One of them chases the other and they move from right to left.
36. *Struggling*: Two actors take part in the scene: both either males or females. They continuously push each other with their arms.
37. *Waving goodbye*: Two actors, one male and the other female, wave to each other.
38. *Beating with a piece of wood*: The actor sits on a sandy area (beach) and beats the sand with a piece of wood.
39. *Carrying with shoulder and hand*: The actor carries some wood on the left shoulder, holding it with a hand. He/she walks from right to left while carrying the load. The background is a grassy area.
40. *Reprimanding a person*: Two actor participate in the scene (one male, the other female). The one on the right reprimands the other using his/her finger.
41. *Biting a banana*: The actor sits in a grassy area, holds a banana in his/her left hand, and bites it.

42. *Fighting with another person*: Two actors participate in the scene: both either males or females. They continually hit each other with the hands and arms.
43. *Washing own body*: The actor stands by the lake and washes his/her arms with the opposite hand.
44. *Foraging*: The actor searches for something with his/her hands in a grassy area.
45. *Stretching own body*: The actor stands in a grassy area and stretches his/her arms towards the sky.
46. *Writing with fingers*: The actor sits in a sandy area (beach) and writes something in the sand with the right index finger.
47. *Charging to attack*: Two actors participate in the scene and face each other. The one on the right makes an attempt with his/her arms to attack the one on the left who remains passive.
48. *Diving*: The actor jumps and dives into a lake (from left to right).
49. *Pointing nearby*: The actor sits in a sandy area (beach) and points to an object (stone) on the sand with his right finger.
50. *Squeezing an orange*: The actor sits in a grassy area, holds an orange in his/her right hand, and squeezes it.
51. *Forbidding with fingers*: Two actors play in the scene (one male, one female). They face each other, and the one on the right moves his/her index finger continuously to left and right (signing "no"). The background is a grassy area.
52. *Wrapping a stone*: The actor sits in a grassy area and wraps a stone into a leaf with his/her hands.
53. *Grasping*: The actor sits in a sandy area (beach) and grasps an object (stone) lying on the ground with his/her right hand.
54. *Carrying on shoulder*: The actor carries some wood on his/her left shoulder without the assistance of the hands. He/she walks from right to left in a grassy area.
55. *Running*: The actor runs in a grassy area from right to left.
56. *Burying in the sand*: The actor sits in a sandy area (beach) and buries an object (stone) into the sand with his/her hands.
57. *Stopping a person*: Two actors participate in the scene (one male, one female), facing each other. The one on the left walks towards the one on the right. The one on the right raises his/her hand and signs "stop". The background is a grassy area.
58. *Pushing a person*: Two actors take part in the scene (one male, one female). They face each other. The one on the right pushes the one on the left (who remains passive). The background is a grassy area.
59. *Kissing a person*: Two actors participate in the scene (one male, one female). They sit in a grassy area and face each other. They bend forward, and the one on the right kisses the other on the cheek.
60. *Throwing and catching a small piece of wood*: Two actors participate in the scene. They face each other. The one on the left throws a piece of wood to the one on the right, who catches it. The background is a grassy area.
61. *Caressing own cheek*: The actor caresses his/her own left cheek with the right hand. The background is a grassy area.
62. *Overtaking an obstacle*: The actor steps over an obstacle (wooden log) with his/her legs. The background is a grassy area.
63. *Touching another person on the shoulder*: Two actors participate in the scene (one male, one female). They face each other. The one on the right touches the right shoulder of the other one with his/her right hand. The background is a grassy area.
64. *Building pyramid from sand*: The actor sits in a sandy area (beach) and builds a pyramid from sand with his/her hands.
65. *Hiding an object behind back*: The actor sits in a sandy area (beach), grasps and hides an object (stone) behind himself/herself.
66. *Washing fruit*: The actor sits on the edge of a lake, holds an apple and washes it.

67. *Carrying with both hands*: The actor walks from right to left in a grassy area and carries a basket with his/her two hands.
68. *Walking*: The actor walks from right to left in a grassy area.
69. *Marching*: The walker marches from right to left in a grassy area.
70. *Rubbing own cheek*: The actor rubs his/her own left cheek with the right hand. The background is a grassy area.
71. *Throwing nearby*: The actor sits in a sandy area (beach) and throws an object (stone) to a nearby location.
72. *Masticating*: The actor sits in a grassy area, holds a peeled banana in his/her hand, and masticates.
73. *People meeting*: Two actors take part in the scene (one male, one female). They face each other, approach, hug, and kiss each other. The background is a grassy area.
74. *Climbing up a tree*: The actor climbs up a tree using his/her feet and hands.
75. *Dancing with another person*: Two actors (one male, one female) dance together in a grassy area.
76. *Getting up*: The actor initially sits in a grassy area, and then gets up.
77. *Crawling*: The actor crawls over a grassy area using his/her knees and elbows.
78. *Spitting a piece of banana*: The actor sits in a grassy area and spits out a piece of banana.
79. *Doing gymnastics with both feet and arms*: The actor does jumping jacks in a grassy area.
80. *Pushing a large object*: The actor pushes a large piece of wood with his/her hands horizontally to the left.
81. *Rolling body sidewise*: The actor rolls his/her body on the ground (towards the camera) in a grassy area.
82. *Walking on hand and knees*: The actor moves his/her body from left to right using hands and knees.
83. *Laughing together with another person*: Two actors (one male, one female) face each other in a grassy area and laugh together.
84. *Carrying with one hand*: The actor walks from right to left and carries a piece of wood in his/her left hand. The background is a grassy area.
85. *Singing a song*: Two actors (one male, one female) face each other, and the one on the right sings a song. The background is a grassy area.
86. *Weighing an object with two hands*: The actor holds a basket and weighs it with his/her hands. The background is a grassy area.
87. *Pointing distantly*: The actor is near a sea, and points towards the water with his/her left arm.
88. *Drinking with hands*: The actor kneels down and drinks water from a fountain with his/her hands. The background is a grassy area.
89. *Massaging another person*: Two actors (one male, one female) participate in the scene. They sit in a grassy area, and the one on the right massages the shoulders of the one on the left.
90. *Drinking with mouth*: The actor kneels down and drinks water from a fountain without using his/her hands. The background is a grassy area.
91. *Measuring height with own body*: The actor stands near a tree and measures his/her height with respect to the tree using his/her hands.
92. *Pinching off piece of banana*: The actor sits in a grassy area, hold a peeled banana in his/her hand, and pinches off a piece of banana with the right hand.
93. *Erasing*: The actor sits in a sandy area (beach) and erases a writing on the sand using his/her hand.
94. *Hugging a person (passive)*: Two actors participate in the scene (one male, one female). The one on the right hugs the other who remains passive. The background is a grassy area.
95. *Speaking with another person*: Two actors (one male, one female) face each other, and the one on the right talks to the other. The background is a grassy area.
96. *Rotating a stone*: The actor sits in a sandy area (beach) and rotates a stone in his/her right hand.
97. *Measuring a short distance with feet*: The actor takes short steps between two pieces of wood to measure the distance between them. The background is a grassy area.
98. *Hugging each other*: Two actors participate in the scene (one male, one female). They hug each other. The background is a grassy area.

99. *Digging a hole with a hand*: The actor sits in a sandy area (beach) and digs a hole with his/her hands.
100. *Throwing far*: The actor stands by a lake and throws an object (stone) into the lake.
